# Effect of Different Doses of Intrathecal Dexmedetomidine as an Adjuvant Combined With Hyperbaric Ropivacaine in Patients Undergoing Cesarean Section

**DOI:** 10.3389/fphar.2020.00342

**Published:** 2020-03-20

**Authors:** Yong-hong Bi, Jia-min Wu, Yan-zhuo Zhang, Rui-qin Zhang

**Affiliations:** Department of Anesthesiology, China and Heilongjiang Key Laboratory for Anesthesia and Critical Care, The Second Affiliated Hospital of Harbin Medical University, Harbin, China

**Keywords:** cesarean section, dexmedetomidine, ropivacaine, somato-visceral sensory block, postoperative analgesia

## Abstract

**Objective:**

In this study, we aim to investigate the effect of different doses of dexmedetomidine as an adjuvant to hyperbaric ropivacaine in spinal anesthesia for cesarean section.

**Methods:**

Seventy-five parturients with American Society of Anesthesiologists (ASA) I or II were anesthetized with intrathecal ropivacaine (12.5 mg) alone (R group) or in combination with dexmedetomidine 3 μg (RD3 group) and 5 μg (RD5 group) to undergo a cesarean section. The anesthetic parameters, postoperative analgesia, stress responses and neonates outcomes were monitored.

**Results:**

The onset time of sensory block to T10, T4, and peak level in the RD3 group and RD5 group were significantly shorter than those in the R group (p < 0.05). The time of the level of sensory block to descend two segments and to T10 in the RD3 group(57.28 ± 16.65 min, 3.87 ± 1.60 h) and RD5 group (71.92 ± 10.10 min, 3.99 ± 1.06 h) were longer than that in the R group (40.64 ± 12.06 min, 1.98 ± 1.01 h) (p < 0.05). The median time of motor blockade to both legs score 3 on the Bromage scale (B3B3) in the RD3 group and RD5 group was shorter than that in the R group (p < 0.001). The time of motor blockade recovery to both legs score 0 on the Bromage scale (B0B0) in the RD5 group (3.6 h) was longer than that in the R group (2 h) or RD3 group (2.2 h) (p < 0.001). Visceral traction response and abdominal muscle relaxation during the operation in the RD3 group and the RD5 group were better than that in the R group. The Visual Analogue Score (VAS) in the 12 h after the operation in the RD3 group (3.30 ± 1.17) and RD5 group (2.80 ± 0.87) was smaller than that in the R group (3.80 ± 1.47) (p < 0.05). The incidence of shivering in the RD3 group and RD5 group was significantly lower than that in the R group (p < 0.05). The postoperative concentrations of c-reactive protein (CRP), interleukin-6 (IL-6) and cortisol in the RD3 and RD5 groups were lower than that in the R group (p < 0.05).

**Conclusion:**

3 µg intrathecal dexmedetomidine as an adjuvant to ropivacaine improved intraoperative somato-visceral sensory block characteristics and postoperative analgesia, alleviated shivering in parturients, and did not prolong the time of motor block or produce any side effects, which makes this dose appropriate for cesarean delivery.

**Cinical Trial Registration:**

ChiCTR, identifier ChiCTR1800014454. Registered 15 January 2018, http://www.chictr.org.cn/edit.aspx?pid=24613&htm=4

## Introduction

Ropivacaine has a lower central nervous and cardiac toxic potential than bupivacaine. Additionally, ropivacaine is less potent, and causes a shorter duration of a motor blockade than bupivacaine (Akerman et al., 1988; Feldman and Covino, 1988). Thus, hyperbaric ropivacaine spinal anesthesia is usually used for cesarean section. However, ropivacaine is insufficient for preventing visceral pain because of its short duration, so analgesia supplementation in the intraoperative period or early analgesic intervention in the postoperative period is often required (Chung et al., 2002).

Several studies have been conducted to elucidate the efficacy of intrathecal ropivacaine with adjuvants such as fentanyl (Chung et al., 2002) and sufentanil (Sun et al., 2001; Chen et al., 2010) in cesarean delivery. But intrathecal opioids will increase nausea and vomiting (Weigl et al., 2017) and itching (Sun et al., 2001; Chen et al., 2010; Sun et al., 2017) that is an uncomfortable experience and prolong the recovery for the parturients after the surgery. Intrathecal dexmedetomidine, an α_2_-adrenergic receptor agonist used as an adjuvant to bupivacaine, has been found to provide better intraoperative nerve blockade than bupivacaine alone as well as prolong postoperative analgesia and alleviate shivering in patients undergoing cesarean section (Bi et al., 2017; He et al., 2017; Xia et al., 2018; Liu et al., 2019). And this minimal dose of intrathecal dexmedetomidine after absorbed into blood and metabolized by the liver may not cause potential fetal toxicity based on the previous studies (Bi et al., 2017; He et al., 2017; Xia et al., 2018; Liu et al., 2019). Zhou et al. found that dexmedetomidine can reduce the traumatic stress response and immune suppression caused by surgery and has a protective effect on the spinal cord in spinal surgery (Zhou et al., 2017).

We hypothesized that dexmedetomidine as an adjuvant added into intrathecal hyperbaric ropivacaine could improve intraoperative blockade conditions in cesarean section, prolong postoperative sensory block, and alleviate shivering with no influence on the motor block and minimal other side effects.

## Materials and Methods

### Study Oversight

Parturients underwent elective cesarean delivery at the Second Affiliated Hospital of Harbin Medical University at Harbin, Heilongjiang Province, China. This study was approved by the institutional ethics committee and registered with www.ClinicalTrials.gov (ChiCTR-TRC-13004622). Informed written consent to participants in this study was obtained from the parturients.

### Participants

A total of 84 parturients with ASA physical status I or II who were scheduled for elective cesarean section under combined spinal-epidural anesthesia were enrolled in this study. The exclusion criteria were as follows: age <18 years; height <150 cm or >180 cm; hypertension; multiple pregnancy; placenta previa; fetal distress in utero; contraindications for intrathecal anesthesia; preoperative heart rate less than 50 bpm with cardiac conduction or rhythm abnormalities; allergic reactions to α_2_-adrenergic agonists or local anesthetics; and an epidural catheter insertion time of more than 3 min.

### Study Outcomes

We first determined whether dexmedetomidine combined with intrathecal hyperbaric ropivacaine could improve block characteristics during cesarean section and prolong postoperative sensory block with no effect on the motor block. Then, we determined which dose of dexmedetomidine was better.

### Study Protocol

The patients were infused with 5 ml/kg of lactated Ringer's solution within 10 min through a 22-gauge cannula inserted in a forearm vein. Electrocardiogram (ECG), blood pressure and pulse oximetry were monitored continuously. Oxygen was routinely given *via* an oxygen mask at a rate of 4 L/min.

Patients were administered combined spinal-epidural anesthesia in the right lateral decubitus position under all aseptic precautions. Epidural puncture was performed in the L_3_–L_4_ intervertebral space using a Durasafe Adjustable BD needle. A computer-generated randomization table was used to divide parturients into three groups: 12.5 mg intrathecal hyperbaric ropivacaine (the R group); 12.5 mg hyperbaric ropivacaine with 3 μg dexmedetomidine (the RD_3_ group); and 12.5 mg hyperbaric ropivacaine with 5 μg dexmedetomidine (the RD_5_ group). All solutions were at room temperature and diluted with 0.9% saline to a final volume of 2.5 ml. The investigator responsible for the random allocation and preparation of spinal anesthetics was not present during surgery or postoperative evaluations. The anesthesiologist responsible for outcome evaluations was blinded to the group assignment. The injection speed in both groups was 0.1 ml/s. The spinal anesthesia needle was withdrawn, and 3 cm of the epidural catheter was placed into the epidural space. Sensory changes were recorded bilaterally along the midclavicular line with a soft needle. If sensory blockade at the T_7_ level was not achieved at the beginning of the operation, an epidural supplement of 2% lidocaine was administered to reach that level, and the parturient was excluded from this study. Motor block was detected using the modified Bromage scale. All anesthesia procedures were performed by the same anesthesiologist. All patients were monitored and observed by another anesthesiologist. The same obstetricians performed all the cesarean sections. The surgical technique was uniform for all patients and included the exteriorization of the uterus. If the patient's pain score on a visual analog scale was ≥3 or the patient requested additional anesthetic despite the T_6_ sensory level being achieved, 5 ml of 2% lidocaine was provided epidurally as an additional anesthetic, repeated every 5 min if necessary. If a visceral traction response occurred during surgery or if the patients experienced any discomfort such as back pain or stomach discomfort after fetal delivery, intravenous fentanyl (0.05 mg) was given immediately. If the pain was not relieved, another 0.05 mg fentanyl was given. Any supplemental doses of lidocaine or fentanyl were recorded. The neonates' physical condition was assessed using Apgar scores at the 1st and 5th min. All patients, regardless of group assignment, received 2 mg morphine through the epidural route at the end of the surgery. Nausea in the parturients was treated with intravenous granisetron (3 mg). Blood collection was completed before anesthesia and after the operation. If the patient's VAS was more than 4 after surgery, the surgeon gave an injection of diclofenac sodium. Promethazine (12.5 mg) was administered if the patient shivered after the operation.

### Measurements

Sensory block was evaluated every 5 min with a pinprick test. Motor block was evaluated with the Bromage scale. the effect and satisfaction of spinal anesthesia, visceral traction response, the quality of abdominal muscle relaxation, and shivering were graded according to **Table 1**.

**Table 1:** Score of measurements.

Score	Bromage scale	Spinal anesthesia	Visceral traction response	Muscle relaxation	Shivering
0	No motor loss				
1	Inability to flex the hip	No pain, good muscle relaxation, quiet, good conditions for the operation, cardiac and lung function and hemodynamic characters stable	No discomfort in stomach or perineum; no nausea, vomiting, or intestinal tympanites	No disturbing muscle strain	Moderate quivering of the faciocervical muscles
2	Inability to flex the knee	Moderate pain, poor muscle relaxation, visceral traction pain, need for sedation, hemodynamic fluctuation	Moderate discomfort in stomach and perineum; no nausea, vomiting, or intestinal tympanites	Disturbing but acceptable muscle strain	Obvious quivering of the neck and upper arms
3	Inability to flex the ankle	Serious pain; poor muscle relaxation; moaning and restlessness; sedative and opioid drugs are needed and do not fully alleviate the pain; operation is completed reluctantly	Serious discomfort in stomach; serious discomfort in perineum; obvious intestinal tympanites; nausea and vomiting that require drug treatment	Unacceptable muscle strain	Obvious quivering of the whole body

The following parameters were observed immediately after the administration of spinal block: onset time of sensory block to T_10_, T_4_, and peak level; time of motor blockade to B_3_B_3_ (both legs score 3 on the Bromage scale); time of sensory block level to descend two segments and to descend to T_10_; time of motor blockade recovery to B_0_B_0_ (both legs score 0 on the Bromage scale); surgery onset time; fetal delivery time, intraoperative supplemental lidocaine dose and time; supplemental fentanyl dose; visceral traction response; abdominal muscle relaxation; patient VAS scores at 2 h, 6 h, and 12 h after surgery; time of first rescue analgesia drug administration; and time to first flatus. Side effects included shivering, nausea and vomiting, hypotension, and pruritus, among others.

The hemodynamic parameters included systolic blood pressure (SBP); diastolic blood pressure (DBP); heart rate (HR); blood oxygen saturation measured by pulse oximetry at 0, 2.5, 5, 7.5, 10, 15, 20, 25, 30, 40, 50, and 60 min after spinal anesthesia; and doses of phenylephrine at 10min, 20 min, and 30min after spinal anesthesia. If the systolic blood pressure decreased by 20% or fell below 100 mmHg, 80 μg phenylephrine was given. Umbilical cord blood gas analysis and Apgar scores were measured after labor. Maternal CRP, IL-6, and cortisol levels were measured from the blood samples of the parturients.

### Statistical Analyses

Data are expressed as the mean ± sd and median (range) as appropriate. Statistical analysis was performed by SPSS 20.0 software. ANOVA and χ^2^ tests were used for analyzing standard characteristics, degree of motor block, sensory block level, maternal side effects, fetal delivery, and postoperative analgesia. Data that were distributed non-normally were analyzed by a Mann-Whitney U test. Categorical variables were analyzed using Fisher's exact test if the number of subjects in any cell of the contingency table was expected to be less than five. The analysis of variance for repeated measures was used to compare hemodynamic characteristics. *P* < 0.05 was considered statistically significant.

## Result

### Demographic and Surgical Characteristics

A total of 84 parturients were enrolled in this study. One patient had a heart rate of less than 50 bpm, 2 patients had multiple pregnancies, 1 patient had fetal distress in utero, 2 patients had an epidural catheter insertion time of more than 3 min, 2 patients failed to obtain blood samples, and 1 patient failed to reach T_7_ blockade during anesthesia who was later changed to general anesthesia. A total of 75 patients were divided into 3 groups, 25 patients in each group completed the experiment. The demographic characteristics of the patients in all 3 groups were comparable regarding age, weight, height, and gestation week (p > 0.05). There were no significant differences in the onset time of operation, fetal delivery time, duration of surgery, or duration of anesthesia among the groups (**Table 2**).

**Table 2:** Demographic and surgical characteristics.

	Group R n = 25	Group RD_3_ n = 25	Group RD_5_ n = 25	*P* value
Age (years)	30.9 ± 5.4	32.6 ± 6.4	31.6 ± 4.8	0.552
Height (cm)	161.8 ± 5.2	162.0 ± 4.6	163.9 ± 4.5	0.234
Weight (kg)	72.8 ± 10.5	75.3 ± 11.3	75.1 ± 11.0	0.663
Gestational week (weeks)	37.8 ± 1.8	38.3 ± 1.2	38.8 ± 1.2	0.069
Onset time of operation (min)	16.5 ± 3.7	14.9 ± 3.8	15.0 ± 3.0	0.213
Fetal delivery time (min)	26.0 ± 3.5	23.7 ± 5.2	23.6 ± 4.7	0.124
Duration of surgery (min)	63.0 ± 9.1	57.2 ± 10.3	59.4 ± 13.9	0.194
Duration of anesthesia (min)	84.6 ± 11.4	76.2 ± 13.8	80.2 ± 15.9	0.103

Data are expressed as mean ± sd, p < 0.05 was considered statistically significant.

### Spinal Block Characteristics and Analgesia

**Figure 1:**
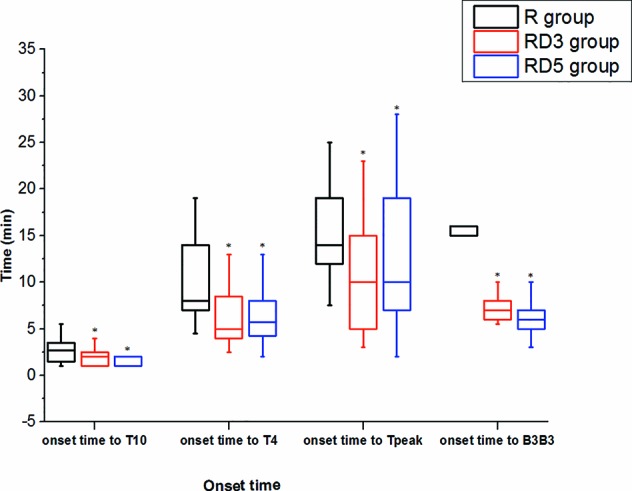
Onset times of sensory and motor block. *p < 0.05 was considered statistically significant compared with the R group.

**Figure 2:**
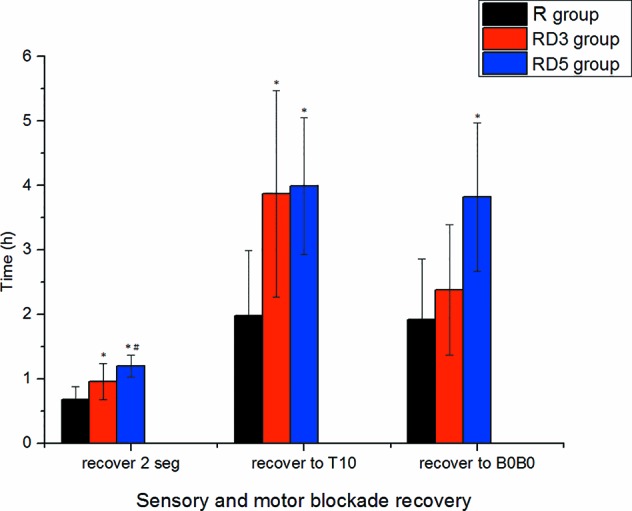
Recovery time of sensory and motor block. *p < 0.05 was considered statistically significant compared with the R group. #p < 0.05 was considered statistically significant compared with the RD3 group.

The onset times of sensory block to T_10_, T_4_, and peak level in the RD_3_ group and RD_5_ group were significantly shorter than those in the R group (p < 0.05). The median time of motor blockade to B_3_B_3_ in the RD_3_ group (7 min) and in the RD_5_ group (6 min) was shorter than in the R group (15 min) signifcantly (p < 0.001) (**Figure 1**). The time of the sensory block level to descend two segments and to descend to T_10_ were longer in the RD_3_ group and RD_5_ group than those in the R group (p < 0.05); furthermore, the time of the sensory block level to descend two segments in group RD_5_was longer than that in group RD_3_ (p < 0.05). Besides, the time of motor blockade recovery to B_0_B_0_ in the RD_5_ group was longer than that in the R group or the RD_3_ group (p < 0.001). However, there was no difference in the recovery time between the R and RD_3_ groups (**Figure 2**).

The median intraoperative rescue dose of lidocaine in the RD_3_ group and the RD_5_ group (both 0 ml) were less than those in the R group (5 ml) (p < 0.05). The median intraoperative rescue dose of fentanyl in the RD_3_ group and RD_5_ group (both 0 mg) was less than that in the R group (0.1 mg) (p < 0.001). There were significantly more patients with no visceral traction response in the RD_3_ group (21 patients, 84%) and RD_5_ group (25 patients, 100%) than those in the R group (2 patients, 8%; p < 0.05). The grade of spinal anesthesia in the RD3 group and RD5 group was more than that in the R group (p < 0.001). 19 patients (76%) in the RD_3_ group and 17 patients (68%) in the RD_5_ group were at in grade 1 of spinal anesthesia, compared to 3 patients (12%) in group R. Satisfaction with muscle relaxation was significantly higher in the RD_3_ and RD_5_ groups than that in the R group (p < 0.05) (**Table 3**).

**Table 3:** Spinal block characteristics.

	R group n = 25	RD_3_ group n = 25	RD_5_ group n = 25	*P* value
Dose of supplemental lidocaine (ml)	5.0(5.0-10.0)	0.0(0.0-5.0)^*^	0.0(0.0-5.0)^*^	**<0.001**
Dose of supplemental fentanyl (mg)	0.10(0.05-0.1)	0.0(0.0-0.25)*	0.0(0.0-0.0)*	**<0.001**
Visceral tract reaction				**<0.001**
1, n(%)	2(8.0)	21(84.0)^*^	25(100.0)^*^	
2, n(%)	21(84.0)	4(16.0)^*^	0(0.0)^*^	
3, n(%)	2(8.0)	0(0.0)^*^	0(0.0)^*^	
Satisfaction with muscle relaxation				**<0.05**
1, n(%)	10(40.0)	20(80.0)^*^	17(68.0)^*^	
2, n(%)	11(44.0)	5(20.0)^*^	8(32.0)^*^	
3, n(%)	4(16.0)	0(0.0)^*^	0(0.0)^*^	
Grade of spinal anesthesia				**<0.001**
1, n(%)	3(12.0)	19(76.0)^*^	17(68.0)^*^	
2, n(%)	21(84.0)	6(24.0)^*^	8(32.0)^*^	
3, n(%)	1(4.0)	0(0.0)^*^	0(0.0)^*^	

Data are expressed as median, *p < 0.05 compared with the R group.

### Analgesia, Side Effects, and Hemodynamics

**Figure 3:**
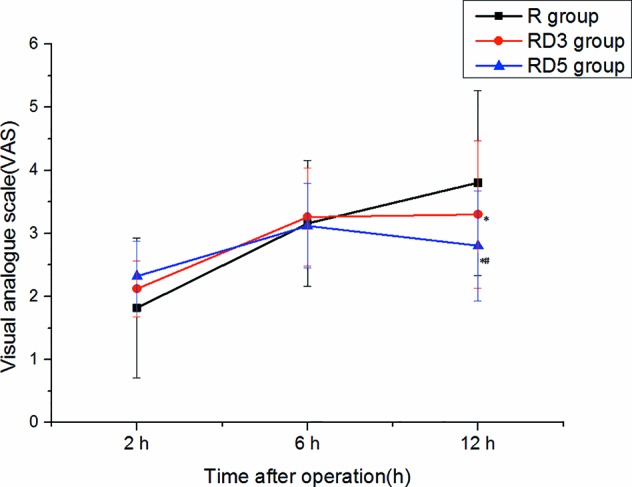
Visual Analogue Score (VAS) pain scores of the three groups. *p < 0.05 was considered statistically significant compared with the R group. #p < 0.05 was considered statistically significant compared with the RD3 group.

There were no significant differences in VAS pain scores among the three groups at the 2 h or 6 h after operation. however, at the 12 h postoperatively, the VAS in the RD_3_ group and RD_5_ group was significantly less than that in the R group (p < 0.05), and the VAS in the RD_3_ group was significantly higher than that in the RD_5_ group (p < 0.05) (**Figure 3**).

The time of postoperative rescue analgesia in group RD_3_ and group RD_5_ was later than that in group R (p < 0.05). The percentage of postoperative rescue analgesia in the group RD_3_ and group RD_5_ was less than that in group R (p < 0.05). There were no significant differences in the time to first evacuation and side effects (nausea and vomiting, pruritus) among the three groups. (p > 0.05) (**Table 4**).

**Table 4:** Postoperative pain and side effects.

	R group n = 25	RD_3_ n = 25	RD_5_ group n = 25	*P* value
Evacuation time (h)	25.4(22.3–27.5)	26.0(25.0–29.0)	26.0(23.5–27.0)	0.365
Postoperative nausea and vomiting, n(%)	2(8)	1(4)	0(0)	0.769
Pruritus, n(%)	2(8)	1(4)	0(0)	0.769
Postoperative rescue analgesia time (h)	12.00(3.75,23.25)	20.50(15.38,25.75)*	26.50(20.75,30.75)*	0.022
Postoperative rescue analgesia, n(%)	12(48)	3(12)*	5(20)*	0.010

Data are expressed as median, *p < 0.05 compared with the R group.

**Figure 4:**
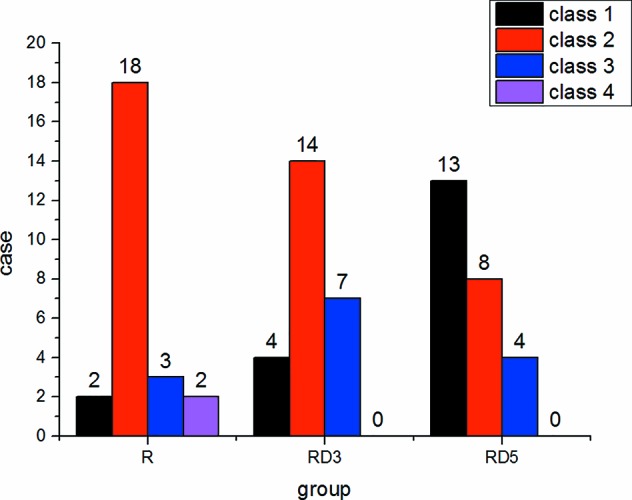
Shivering in the three groups.

The incidence of shivering in the RD_3_ group and RD_5_ group was significantly lower than that in the R group (p < 0.05) (**Figure 4**).

The dose of phenylephrine within the first 10 min after spinal anesthesia in the RD_3_ and RD_5_ groups was more than that in the R group (p < 0.05). No significant difference was observed in the dose of phenylephrine within the first 20 min or 30 min after spinal anesthesia among the three group. (**Table 5**).

**Table 5:** Phenylephrine dose after anesthesia.

	**Group R** **n = 25**	**Group RD_3_** **n = 25**	**Group RD_5_** **n = 25**	***P* value**
Phenylephrine dose at 10 min (ml)	3.20 ± 1.89	5.24 ± 2.45^*^	5.52 ± 3.48^*^	**0.006**
Phenylephrine dose at 20 min (ml)	6.70 ± 3.58	7.90 ± 3.72	9.26 ± 5.89	0.142
Phenylephrine dose at 30 min (ml)	9.22 ± 4.59	9.46 ± 4.39	11.62 ± 7.61	0.270

Data are expressed as mean ± sd, *p < 0.05 compared with the R group.

**Figure 5:**
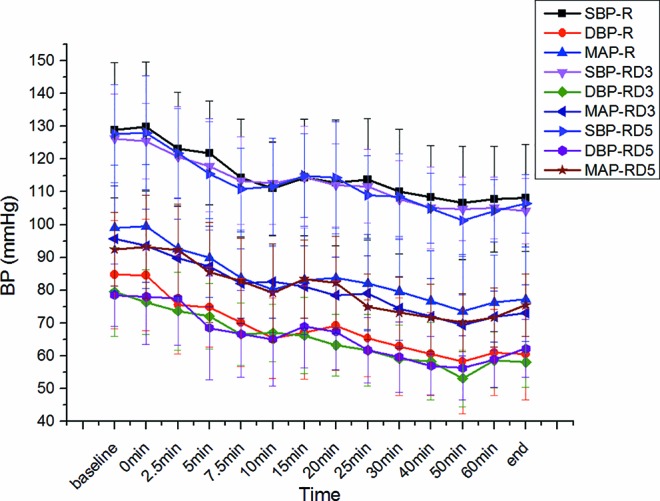
Systolic blood pressure (SBP), diastolic blood pressure (DBP), or MAP among the three groups.

**Figure 6:**
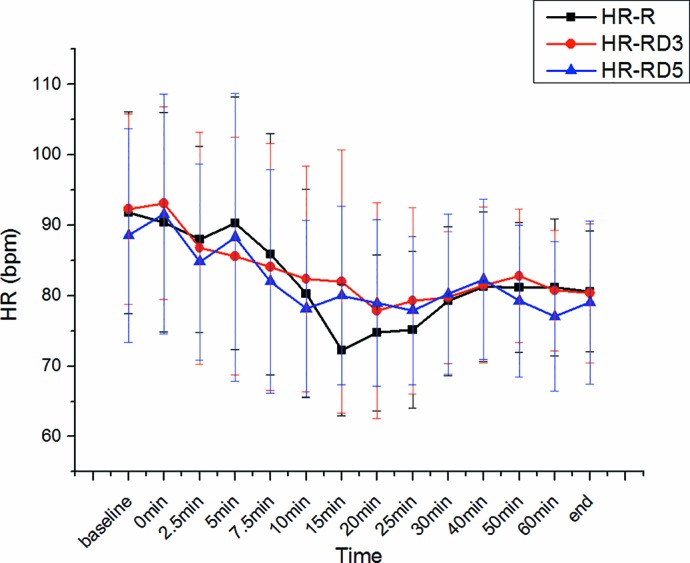
Heart rate among the three groups.

There was no significant difference in SBP, DBP, MAP, and heart rate among the three groups. (**Figures 5** and **6**).

### Fetal Characteristics

There were no significant differences in neonatal Apgar scores or analyses of umbilical cord blood gas, which included pH, partial pressure of oxygen (PO_2_), partial pressure of carbon dioxide(PCO_2_), glucose (Glu), and lactate (Lac) (p > 0.05) (**Table 6**).

**Table 6:** Neonatal Apgar score and umbilical cord blood air analysis.

	**Group R** **n = 25**	**Group RD_3_** **n = 25**	**Group RD_5_** **n = 25**	***P* value**
PH	7.34(7.32, 7.37)	7.36(7.33, 7.38)	7.34(7.32, 7.37)	0.464
PO_2_	31.0(21.0, 37.5)	32.0(26.0, 39.0)	30.0(22.5, 35.0)	0.491
PCO_2_	46.0(39.0, 48.5)	43.0(39.0, 46.5)	43.0(39.0, 47.0)	0.820
Glu	3.60(3.25,4.00)	3.60(3.15, 3.85)	3.80(3.35, 4.25)	0.288
Lac	1.60(1.30,1.95)	1.50(1.20, 1.75)	1.90(1.60, 2.70)	0.401
1 min Apgar	8.5(8.0, 9.0)	9.0(8.0, 9.0)	9.0(8.0, 9.0)	0.061
5 min Apgar	9.0(9.0, 10.0)	10.0(10.0, 10.0)	10.0(10.0, 10.0)	0.368

Data are expressed as median, p < 0.05 was considered statistically significant.

### Stress Response

There was no difference in baseline (preoperative) maternal CRP, IL-6, or cortisol levels among groups. However, the postoperative maternal CRP, IL-6, and cortisol levels in the RD_3_ group and RD_5_ group were lower than those in the the R group (p < 0.05) (**Table 7**).

**Table 7:** CPR, IL-6, cortisol pre and postoperation.

	**Group R** **n = 25**	**Group RD_3_** **n = 25**	**Group RD_5_** **n = 25**	***P* value**
Preoperative CRP concentration (µg/L)	1,472.81 ± 110.33	1,550.82 ± 136.77	1,575.27 ± 104.72	0.067
postoperative CRP concentration (µg/L)	2,844.16 ± 136.45	2,285.24 ± 191.53*	2,428.22 ± 214.79^*^	<0.001
Preoperative IL-6 concentration (ng/L)	50.14 ± 6.70	49.41 ± 6.98	51.61 ± 5.17	0.633
Postoperative IL-6 concentration (ng/L)	107.08 ± 6.61	87.30 ± 4.71*	87.11 ± 5.41^*^	<0.001
Preoperative cortisol concentration (µg/L)	85.80 ± 10.25	85.84 ± 10.11	93.35 ± 10.42	0.089
Preoprative cortisol concentration (µg/L)	160.14 ± 20.14	125.25 ± 11.17*	125.69 ± 6.61^*^	<0.001

Data are expressed as mean ± sd, *p < 0.05 compared with group R.

## Discussion

In this prospective, randomized, double-blind, placebo-controlled study, we observed that the addition of 3 μg or 5 μg dexmedetomidine to hyperbaric ropivacaine for spinal anesthesia in parturients hastened the onset of sensory and motor block, prolonged the time of sensory block, decreased visceral traction response, improved muscle relaxation, enhanced postoperative analgesia, and alleviated shivering without increasing other side effects. We were pleasantly surprised that RD_3_ prolonged the time of the sensory block and did not prolong the time of motor block.

These findings were in concordance with the results of Al-Mustafa et al. (2009), who concluded in their studies that intrathecal dexmedetomidine as an additive to bupivacaine hastened the onset of sensory block and accelerated the spread of sensory and motor block across spinal levels. However, the onset times observed in the study conducted by Mahendru et al. (2013) did not change whether dexmedetomidine was added to the standard hyperbaric bupivacaine. The different results in our study can be attributed to different definitions of onset time (the T_8_ dermatome vs T_10_ in our study) and the fact that the sensory block level of limb surgery is lower in the spine than that in cesarean section.

We observed that intrathecal dexmedetomidine was associated with a shortened latency of motor block and a prolonged motor block effect. Our results were similar to those of the studies by Mahendru et al. (2013), Safari et al. (2016), and Gupta et al. (2011) who observed the intrathecal dexmedetomidine with bupivacaine. In our study, the time of motor blockade recovery to B_0_B_0_ was longer in the RD_5_ group (3.82 ± 1.15 h) than in the RD_3_ group (2.38 ± 1.01 h) or the R group (1.92 ± 0.94 h) in our study. Intrathecal dexmedetomidine (3 µg) do not prolong motor block duration. And early mobilization enhances postpartum recovery and shortens the period of hospitalization (Hedderson et al., 2019; Macones et al., 2019).

Intrathecal dexmedetomidine provided satisfactory muscle relaxation and reduced the reaction to visceral traction. Patients sometimes complain of abdominal pain, nausea and vomiting during cesarean section performed under spinal anesthesia. The pain is thought to be visceral pain, transmitted by unmyelinated C fibers (Alahuhta et al., 1990). Several clinical studies have shown that increasing doses of local anesthetics (Pedersen et al., 1989; Lu et al., 2018), epidural fentanyl (Ishiyama et al., 2001), and an intrathecal combination of clonidine and fentanyl (Naithani et al., 2015) can improve analgesia during cesarean section under spinal anesthesia. These treatments have been performed effectively in practice. In our study, intrathecal dexmedetomidine significantly decreased the visceral traction reaction and enhanced the patients' comfort.

In our study, there was no significant difference in SBP, DBP, MAP, and HR among the three groups. Intrathecal dexmedetomidine has a substantial hemodynamic effect, causing hypotension and bradycardia (Kamibayashi and Maze, 2000; Hall et al., 2001). In addition, it hastens the onset of motor and sensory block. Intrathecal local anesthetics blocks the sympathetic outflow and reduce the hemodynamic parameters during the intraoperative period (Mantouvalou et al., 2008). However, this short-term lower blood pressure did not affect the Apgar scores or the umbilical arterial pH.

The α_2_-adrenergic agents also have anti-shivering properties, as observed by Miao et al. (2018). In our study, we observed the same effect of intrathecal dexmedetomidine. We also did not observe any other side effects, such as bradycardia, in the RD_3_ or the RD_5_ group. The reason may be that we used small doses of intrathecal dexmedetomidine (3 μg, 5 μg) in our study; these doses had been previously supported by Mohamed et al. (2012).

Nasr and Abdelhamid studied the effect of caudal dexmedetomidine versus fentanyl and bupivacaine on the stress response and postoperative analgesia in pediatric cardiac surgery and found that dexmedetomidine attenuated the stress response and improved analgesia (Nasr and Abdelhamid, 2013). Kang reported that dexmedetomidine administration during surgery reduced the intraoperative and postoperative secretion of cytokines, including the proinflammatory cytokines tumor necrosis factor-α, interleukin-1β and IL-6, and the anti-inflammatory cytokines IL-4 and CRP (Kang et al., 2013). Nour EM also found that epidural administration of dexmedetomidine as an adjunct to bupivacaine inhibited the increase in plasma IL-6 (Nour et al., 2013). Similarly, in this study, the postoperative IL-6 and CRP levels in the RD_3_ group and RD_5_ group were lower than those in the R group after surgery. Preoperative anxiety, fear, sleeplessness, anesthesia, surgery, and postoperative pain can elevate cortisol levels dramatically. We found that cortisol secretion was suppressed by a low dose of dexmedetomidine in our study.

All patients, regardless of group assignment, received 2 mg morphine through the epidural route at the end of the surgery. This may be the reason for the lack of difference in VAS scores 2 h and 6 h after surgery. And the pain the patient suffered at 6h is more severe than that at 12 h after the operation. At 12 h after the surgery, the effect of epidural morphine was diminished, and the long-acting analgesic effect of dexmedetomidine was shown. so the VAS in the RD_3_ group and RD_5_ group was lower than that in the R group at 12 h postoperatively.

Local anesthetics act by blocking sodium channels, but α_2_-adrenoceptor agonists act by binding to presynaptic C-fiber and postsynaptic dorsal horn neurons. Intrathecal α_2_-adrenoceptor agonists exert their analgesic effect by depressing the release of C-fiber transmitters and by hyperpolarizing postsynaptic dorsal horn neurons (Fairbanks and Wilcox, 1999; Nguyen et al., 2017). It may be an additive or synergistic effect secondary to the different mechanisms of action of local anesthetics and α_2_-adrenoceptor agonists as studied by Salgado et al. (2008). The antinociceptive effect accounts for the prolongation of the sensory block when an α_2_-adrenoceptor agonist is added to spinal anesthetics. The prolongation of the motor block from spinal anesthetics may result from the binding of α_2_-adrenoceptor agonists to motor neurons in the dorsal horn (Yaksh and Reddy, 1981; Harada et al., 1995). In addition, the direct antinociceptive action of α_2_-adrenoceptor agonists, controlling somatic and visceral pain can further contribute to the prolongation of analgesia duration (Asano et al., 2000; Mahendru et al., 2013).

## Conclusion

3 µg intrathecal dexmedetomidine as an adjuvant to ropivacaine improved intraoperative somato-visceral sensory block characteristics and postoperative analgesia, alleviated shivering in parturients and did not prolong the time of motor block and produce any side effects, which makes this dose appropriate for cesarean delivery.

## Data Availability Statement

All datasets generated for this study are included in the article/supplementary material.

## Ethics Statement

The studies involving human participants were reviewed and approved by the institutional ethics committee of Harbin Medical University at Harbin, Heilongjiang Province, China, and registered with www.ClinicalTrials.gov (ChiCTR-TRC-13004622). The patients/participants provided their written informed consent to participate in this study.

## Author Contributions

Y-ZZ conceived the research. Y-HB performed the spinal-epidural anesthesia. J-MW monitored and observed the parturients. R-QZ analyzed the data. Y-HB, J-MW, and Y-ZZ wrote the manuscript. Y-HB, J-MW, R-QZ, and Y-ZZ revised the manuscript. All authors approved the final version of the manuscript.

## Conflict of Interest

The authors declare that the research was conducted in the absence of any commercial or financial relationships that could be construed as a potential conflict of interest.
